# Pulmonary arteriovenous malformation causing lung sequestration and media infarction: a case report

**DOI:** 10.3389/fmed.2025.1490820

**Published:** 2025-02-11

**Authors:** Raffaella Griffo, Laura V. Klotz, Lena Brendel, Romina Rösch, Benedikt Niedermaier, Kai Schlamp, Mark Kriegsmann, Martin M. Eichhorn, Hauke Winter

**Affiliations:** ^1^Thoraxklinik, Department of Thoracic Surgery, Heidelberg University Hospital, Heidelberg, Germany; ^2^Translational Lung Research Center (TLRC), Heidelberg, Germany; ^3^Thoraxklinik, Department of Radiology, Heidelberg University Hospital, Heidelberg, Germany; ^4^Thoraxklinik, Department of Pathology, Heidelberg University Hospital, Heidelberg, Germany

**Keywords:** lung malformation, thoracic surgery, pulmonary arteriovenous malformation, hereditary hemorrhagic telangiectasia, lung disease, case report

## Abstract

Pulmonary arteriovenous malformations (PAVMs) are rare vascular malformations of the lungs. Direct communication of pulmonary arteries to pulmonary veins is the defining characteristic allowing venous blood to bypass the pulmonary capillary system and avoiding an efficient oxygenation process. The complexity of the pathology lies not only in the variety of its manifestations, but also in the choice of the most appropriate and effective treatment. We present a case of a complex PAVM associated with a persistent foramen ovale, with stroke as the onset symptom. Despite timely multidisciplinary treatment of the malformation, a potentially fatal pulmonary complication occurred, highlighting the critical importance of early, interdisciplinary management and ongoing follow-up of PAVMs, particularly in preventing life-threatening outcomes.

## 1 Introduction

Pulmonary arteriovenous malformations (PAVMs) are characterized by pathological angioarchitecture, with an abnormal connection between pulmonary arteries and veins. This anatomic right-to-left shunt permanently impairs gas exchange and the filtration process of systemic venous blood, leading to possible life-threatening complications such as hypoxemia, sequestration of the lung, and vascular rupture with severe bleeding (1).

The exact prevalence of PAVMs in the general population remains unknown. Idiopathic forms are rarely reported in the literature. PAVM may be present from birth; pulmonary symptoms in children are rare and are reported in only about 15% of affected children (2). These malformations occur mainly in the female sex. Approximately 70% of cases are associated with hereditary hemorrhagic telangiectasia (HHT), also known as Osler-Weber-Rendu (OWR) disease (2).

It is an autosomal dominant transmitted disease characterized by systemic vascular dysplasia. The genetic disorder is based on dysregulation of the transforming growth factor-ß (TGF-ß)/bone morphogenic protein (BMP) pathway and leads to the development of vascular abnormalities. The most common genetic mutations associated with HHT are those affecting endoglin (ENG) or activin A receptor type 1 (ACVRL1 or ALK1), which account for over 85% of cases. Mutations in mothers against decapentaplegic homolog 4 (SMAD4), associated with juvenile polyposis, and in growth differentiation factor 2 (GDF2, also known as BMP-9), are much rarer (3, 4).

Identifying at least three of the four Curacao clinical criteria enables a definitive diagnosis of PAVM: (1) cutaneous or mucosal telangiectasias, (2) positive family history, (3) epistaxis, (4) documented arteriovenous malformations (e.g., in the lung, liver, brain) (4).

We present a case of a young woman with complex PAVM, associated with a persistent foramen ovale, with stroke as the initial symptom. Early genetic testing ruled out an association with the more common forms of HHT, confirming it as a rare variant of the disease. Despite timely, multidisciplinary treatment of the pulmonary malformation, the patient developed severe pulmonary bleeding 3 months after surgery.

This case underscores the complexity of the disease and its potential to lead to catastrophic sequelae, emphasizing the importance of prompt, combined management, where surgery plays an indispensable role, particularly for complex forms. It also highlights the need for continued support for these patients after treatment, including physical and cardiorespiratory rehabilitation, and the prevention of complications related to recanalisation through regular follow-up (3, 5–8).

## 2 Case presentation

An 18-year-old female was admitted to the hospital because of aphasia, hemiparesis and hemihypoesthesia on the right side. Her medical history included only an episode of short-term visual impairment bilaterally 2 years earlier. There was no history of oral or nasal telangiectasia. She was a non-smoker and physically active, with no PAVM precedent in her family history.

**Figure 1:**
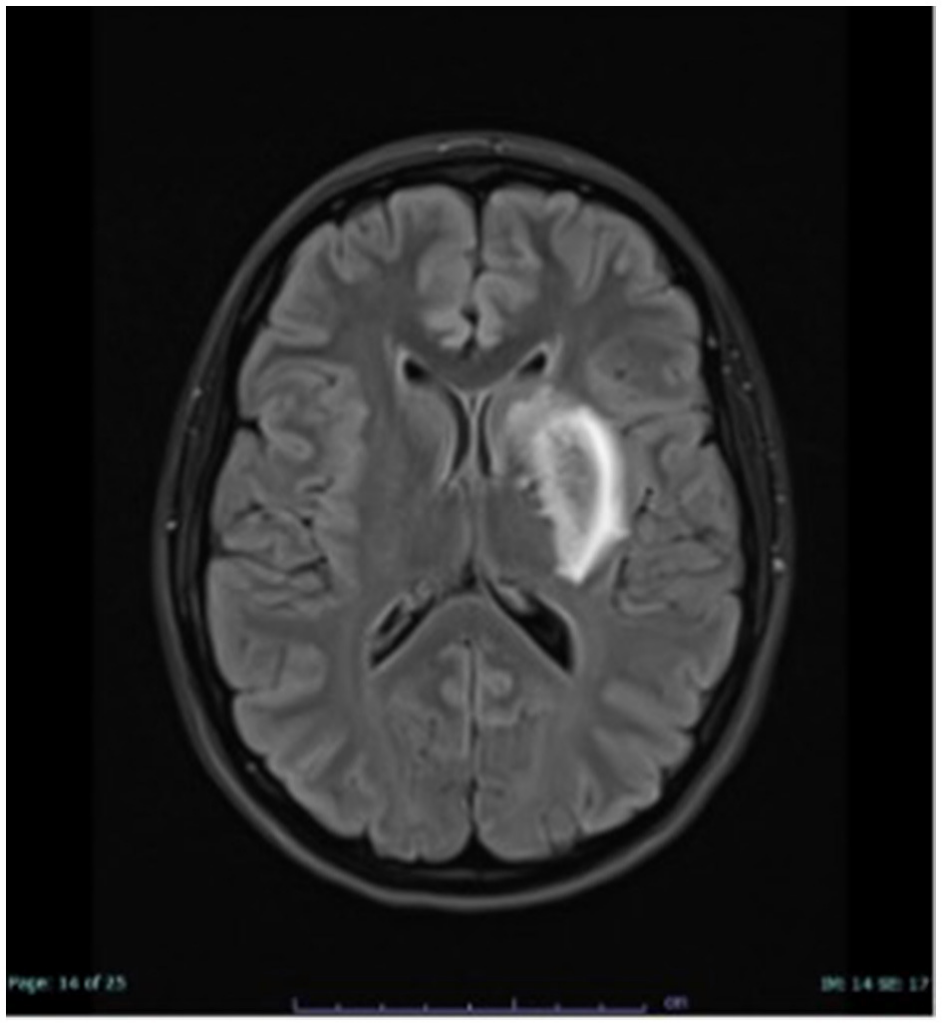
MRI examination of the head, FLAIR hyperintense demarcated subacute ischaemia in the left basal ganglia.

Brain magnetic resonance imaging (MRI) revealed an incipient infarct demarcation in the left arteria cerebri media territory (Figure 1). CT-angiography confirmed an occlusion of the left M1 segment of this artery. Since HHT was suspected, systemic thrombolytic therapy was not used, and mechanical thrombectomy was performed. After the procedure, symptoms resolved, and significant clinical improvement was observed. Secondary prophylaxis with aspirin was initiated.

During the hospital recovery period, a thorough diagnostic workup was conducted. Transthoracic contrast echocardiography (TTCE) revealed persistent foramen ovale with marked contrast crossover on the Valsalva maneuver. Computed tomography (CT) of the chest revealed an extensive PAVM in the right lung.

The patient was referred to the thoracic surgeons for PAVM treatment and discharged from the neurology clinic 8 days later.

After discharge, only the two most common phenotypes of HHT disease (mutations in the genes for ENG or ALK1) were tested and excluded by genetic screening.

Two months later, the patient was admitted to the thoracic surgery department of the Thoraxklinik.

She presented with the following key clinical history and diagnostic imaging:

1. Short-Term Visual Impairment: apart from a spontaneous single episode of short-term visual impairment in the past, there were no reported neurological symptoms such as migraine or any history of bleeding, particularly epistaxis;
2. Cerebral Infarction: two months before admission, the patient experienced a cerebral infarction, accompanied by hemiparesis and right-sided hemihypoesthesia;
3. Radiological Investigations: diagnostic imaging performed up to that point included MRI and angiography of the brain, as well as chest CT and TTCE;
4. Genetic Testing: genetic testing was incomplete and did not confirm a diagnosis of HHT;
5. Physical Examination: no signs of cutaneous or mucosal telangiectasias. Therefore, in the absence of other supporting findings, and given the presence of pulmonary arteriovenous malformations alone, the patient did not meet the full Curacao criteria for a clinical diagnosis of HHT.

**Figure 2:**
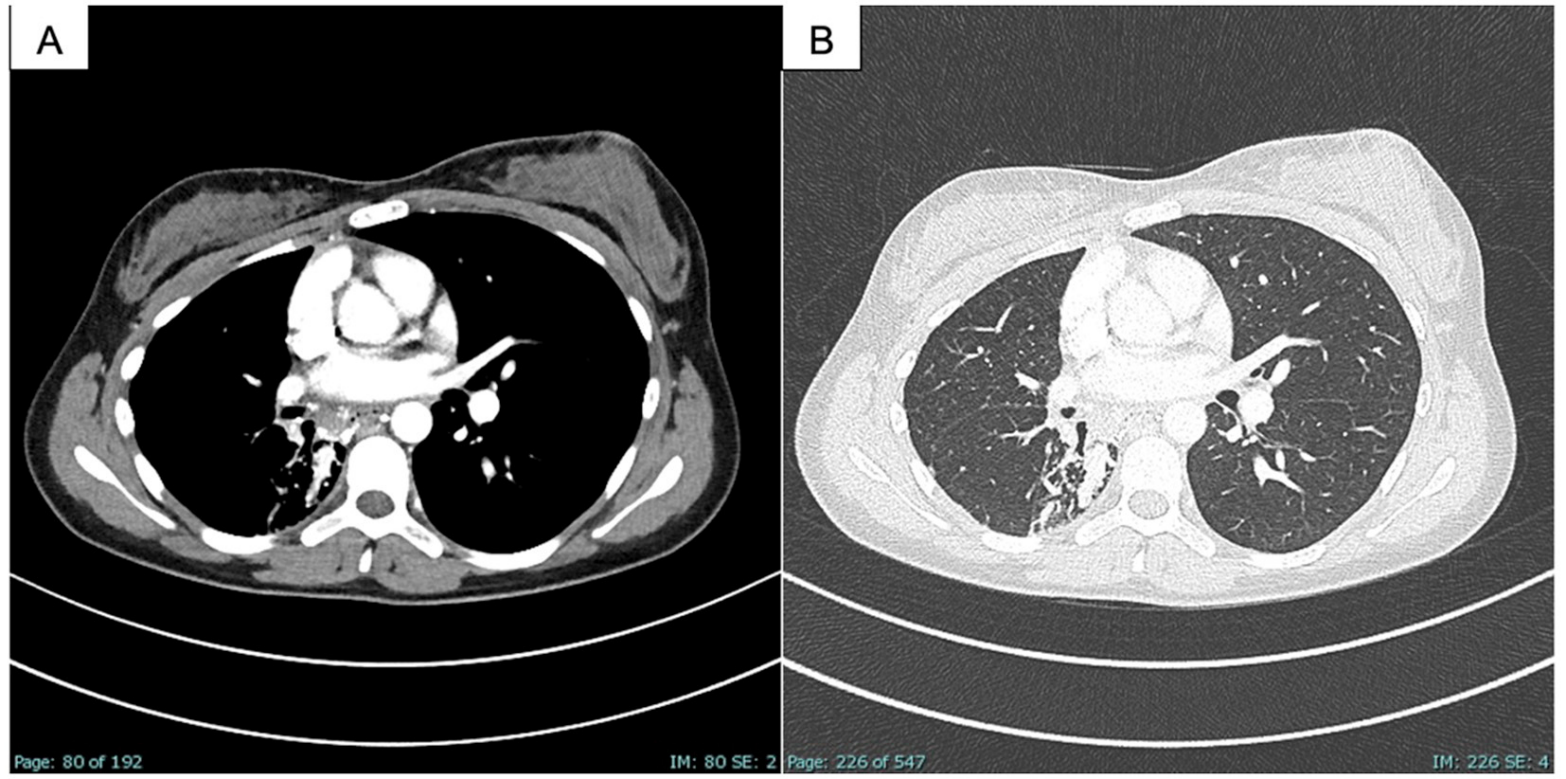
CT scan of the chest, PAVM part in the right middle and lower lobe as well as embolism in the afferent bronchial arteries, **(A)** soft tissue window; **(B)** lung window.

**Figure 3:**
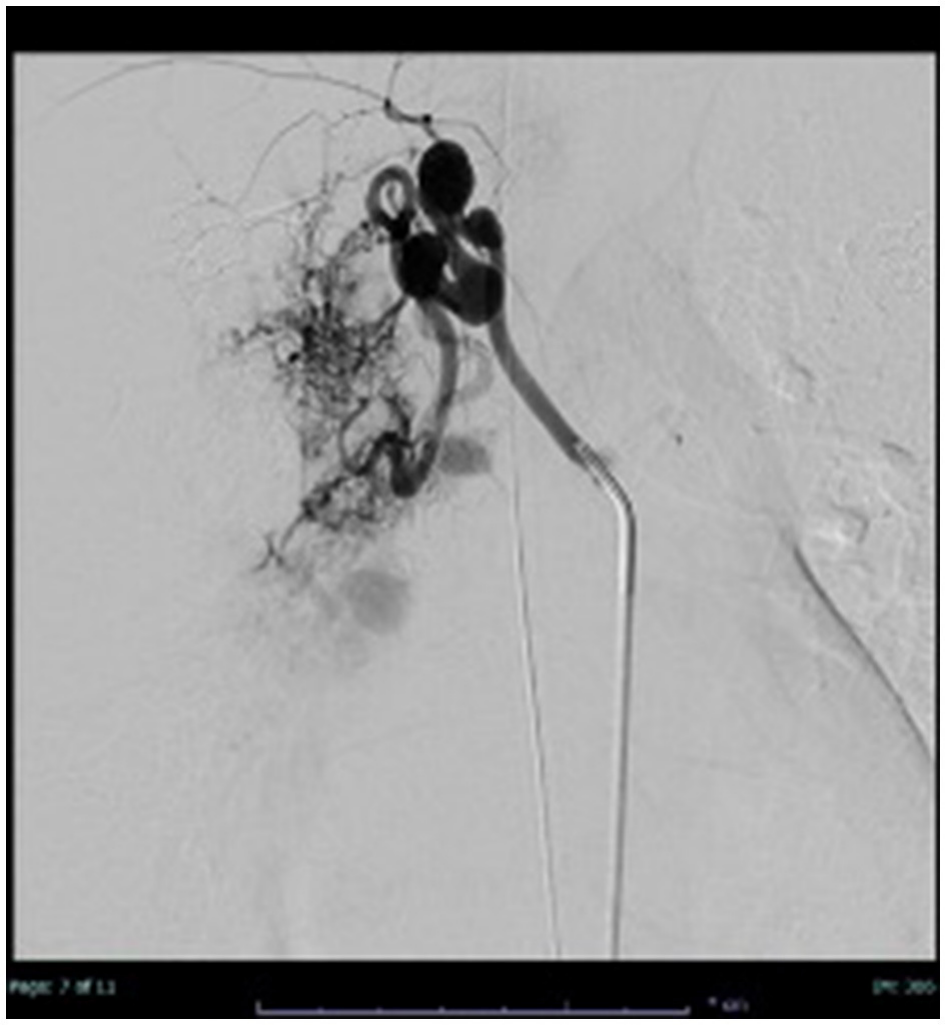
Thoracic angiography: application of contrast medium into a dilated bronchial artery and partial contrasting of the PAVM.

A careful examination of the previously performed chest CT scan showed PAVM with hypotrophy of the right pulmonary artery and decreased perfusion of the middle and lower lobes of the lung (Figure 2). Contrast-enhanced pulmonary angiography confirmed a complex vascular malformation with an aortopulmonary shunt through a dilated bronchial artery and major aortopulmonary collateral arteries (MAPCAs) draining into the superior pulmonary vein (Figure 3).

After interdisciplinary discussion, surgical intervention was scheduled to prevent further thromboembolic events. To reduce the risk of intraoperative bleeding, interventional radiologists performed partial bronchial artery embolization with detachable balloon.

Two days later, the patient underwent surgery via anterolateral thoracotomy. Intraoperatively, isocyanide green was administered venously to visualize spontaneous blood flow. Using blue light, spontaneous perfusion of the upper lobe was demonstrated. No blood flow could be identified in the middle lobe segments IV and V. As expected, perfusion of isocyanide green was observed only in the upper lobe with a delayed influx via upper lobe segment II. Because of the confirmed lack of arterial perfusion, the decision was made to perform a lower bilobectomy.

**Figure 4:**
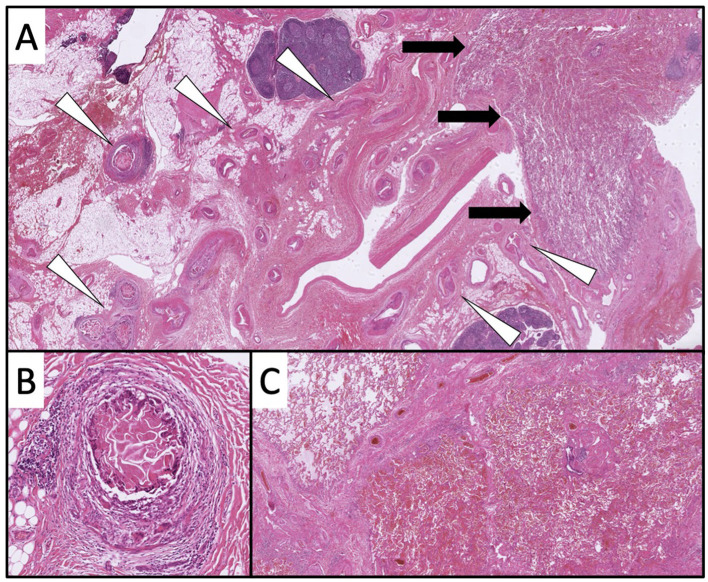
In the overview, a vascular volume (white triangles) in close proximity to the lung parenchyma is recognizable [black arrows; **(A)** 10× magnification, HE]. Individual vessels show intravascular emboli [**(B)** 100× magnification, HE]. In the lung parenchyma, clear hemorrhages are partially detectable [**(C)** 20× magnification, HE].

Histological examination of the right middle and lower lobes revealed numerous vessels, including ectasis, intravascular emboli and clear hemorrhages consistent with pulmonary sequestration (Figure 4). The postoperative course of healing was uneventful.

Three months after discharge, the patient was readmitted with haemoptysis. Vital signs were typical. The complete blood count was unremarkable. Physical examination revealed no relevant findings. CT of the chest and bronchoscopic examination confirmed active bleeding from the right upper lobe. Due to severe bleeding, interventional radiologists rule out the possibility of performing another embolisation. The patient immediately underwent anterolateral thoracotomy with resection of the remaining upper lobe.

The postoperative course was ordinary. Considering the diagnosis, the severe complication recently encountered, and the potential future complications associated with the condition, psychological support was offered to both the patient and her parents. However, this support was declined during hospitalization. The patient's primary request was to be discharged at the earliest opportunity and to resume a normal life.

In consultation with the neurologists who had previously treated the patient, it was advised that she continue secondary prophylaxis with aspirin. Due to the elevated thromboembolic risk, the use of hormonal contraceptives was contraindicated, and the patient was referred for a gynecological consultation. We also recommended completing genetic testing to rule out the possibility of a rare form of HHT linked to mutations in SMAD4 or BMP-9. Additionally, the patient was advised to explore cardiac treatment options for the persistent foramen ovale.

Neuropsychological support, physiotherapy, and ergometry were also recommended as part of her ongoing care. In light of the physical and, particularly, psycho-emotional strain endured in recent months, the patient specifically requested a rehabilitation phase to facilitate her recovery and to allow for further investigations at a later stage.

Since the last follow-up, no additional complaints have been reported.

## 3 Discussion

PAVMs are rarely occurring pathological lung malformations. The etiology and exact pathogenesis of PAVM still remains unknown (9). The most significant correlation with genetic pathology is with HHT (2). In contrast, acquired forms are uncommon and predominantly associated with cancer, cardiothoracic surgery after-effects, infection, amyloidosis, chronic thromboembolic disease, and cancer (3). A frequent coexistence of PAVM and pulmonary hypertension (PH) has also been observed, with reported rates between 1.5% and 13%. In these cases, treating PAVM could increase pulmonary vascular resistance and aggravate the course of PH (10). In our patient, the absence of mutations associated with HHT1 and HHT2 suggests that it may be an isolated congenital form or a rare HHT type 3 or 4 variant.

The difficulty in assessing this disease lies in the variety of its clinical manifestations. In many patients, the disease is asymptomatic. Potentially fatal complications such as massive hemoptysis or hemothorax are rare and are observed in less than 10% of patients (5, 6). Some patients suffer from asymptomatic hypoxemia, but this is often due to hemodynamic compensation even when SaO2 is significantly reduced (<85%) and does not limit their physical activity. Other possible symptoms may include cyanosis (usually masked by anemia) and drumstick fingers (9, 11). The size of the right-to-left shunt determines the degree of hypoxia, and this, combined with the heightened susceptibility to embolic phenomena, underpins the increased risk of neurological complications in these patients. These can include migraine, transient ischemic attack (TIA), stroke, cerebral abscess, and hemorrhage (12–14). In a follow-up study of 155 patients with PAVM, it was found that up to one-third had a history of TIA or ischemic stroke (15). In this case, the patient's isolated episode of temporary vision loss may have been an early warning sign of the disease. However, it was not until 2 years later, when the patient had the stroke, that the true cause was identified.

In patients suspected of having PAVM, TTCE should be performed. This examination allows assessment of the extent of the AV shunt by identifying bubbles in the left atrium and allowing quantification of the right-to-left shunt by contrast administration (9, 16, 17). Intravenously administered agitated saline contrasts air microbubbles in the right heart. The air microbubbles are short-lived and diffuse into the lungs as they travel through the pulmonary circulation. Therefore, the microbubbles enter the left heart only if there is an intracardiac or extracardiac pulmonary arteriovenous shunt from right to left (9, 17). Thoracic CT with thinly sliced 1–2 mm reconstructions establishes the diagnosis. CT with contrast may be beneficial in detecting extrapulmonary vascular abnormalities and planning treatment for PAVM by providing a more detailed assessment of their angioarchitecture. Contrast-enhanced pulmonary angiography is also essential for guiding treatment decisions (4, 9, 11, 18). In this case, chest CT scan and pulmonary artery angiography were crucial in determining the location and extent of the lesion, playing an essential role in treatment planning.

Additionally, genetic testing should be performed to identify or exclude a gene mutation, providing valuable information for patient management and prognostic evaluation (3).

PAVMs can be classified as unilateral or bilateral, single or multiple, and simple or complex based on their distribution and angioarchitecture. In a “simple” PAVM, a single segmental arterial malformation is present. In contrast, in “complex” PAVM, as in the case of our patient, two or more pulmonary artery branches are involved (19, 20). Complex forms are the most challenging to treat successfully. The primary difficulty lies in selecting the optimal treatment approach, minimizing the risk of complications, and ensuring patient adherence to long-term follow-up care.

Transcatheter embolisation remains the treatment of choice. Cases of embolisation in treating PAVM have been reported since 1977 (10). The main techniques involve the use of coils or detachable balloons. Both are based on using angiography to identify the malformation and monitor the success of the treatment. In the case of embolisation with steel coils, it may be necessary to use up to 10 coils for a single PAVM. The detachable balloon embolotherapy technique also involves the occlusion of the neck of the PAVM (21). By insufflating the balloon with radiopaque contrast material, the vessel gets occluded. However, the advantage of this technique is the ability to deflate and replace the balloon if necessary (21, 22). This was the approach used by our interventional radiologists during the patient's pre-operative phase.

These techniques involve risks. Although the embolisation of a simple PAVM is technically more straightforward and less time-consuming, it has a higher risk of paradoxical embolisation (23, 24). Furthermore, post-embolisation complications seem to increase when the afferent artery has a diameter >8 mm. For this reason, therapeutic options should be discussed on an interdisciplinary basis. In cases where one or more malformations with a diameter of more than 2–3 mm are present or in patients with a measurable increase in the size of the AV shunts surgical therapy should be performed (25, 26). Since interventional treatment attempts fail more frequently and complex PAVMs cannot always be treated adequately by interventional means, surgical therapy has become of great importance in the treatment of this clinical condition. A Japanese study by Nagano et al. compared surgery and percutaneous transcatheter embolisation in treating PAVM. Despite the higher incidence of postoperative complications, surgery demonstrated a significantly lower rate of reintervention for PAVMs, suggesting that it offers greater curative potential compared to embolotherapy (7).

In our patient, the primary goal of the treatment during the initial admission was to prevent the progression of PAVM and reduce the risk of serious complications. The decision to use a combined treatment approach with initial embolisation to reduce the risk of intraoperative bleeding, followed by surgical resection, was the most proper. During the second admission, however, due to acute hemoptysis, in consultation with the interventional radiologists emergency surgery was chosen. Numerous studies described in the literature highlight the life-saving role of surgery in managing serious complications, such as hemothorax, paradoxical emboli, or recanalisation after embolotherapy (3, 5–7, 26).

The limitation of our therapeutic approach may lie in the initial treatment. Intraoperatively, given the good perfusion of the upper lobe, we opted for an inferior bilobectomy to avoid pneumonectomy. At the time of discharge, a thoracic MRI check-up 3 months after surgery was recommended. Upon critical assessment of the case, we recognize that additional diagnostic tests or even a new transcatheter examination could have been performed before discharge to assess the condition of the remaining lobe (27). However, this does not suggest we could have prevented the pulmonary complication, and no such data are available in the literature.

The patient's post-operative course in the intensive care unit during both hospitalisations was uneventful. In cases involving critical respiratory conditions, emerging therapeutic strategies should be considered, such as the implementation of advanced airway clearance protocols for mechanically ventilated patients, as well as neuromodulation techniques that may influence extubation outcomes (28, 29).

Recognizing the substantial benefits of exercise in enhancing cardiorespiratory capacity and modulating the immune system's inflammatory response, our patient engaged in daily physiotherapy and respiratory activities during the postoperative course (8, 30, 31). Furthermore, she was advised to pursue a structured physical rehabilitation program following discharge.

Adherence to long-term follow-up is also a key aspect in the management of patients with this condition. There are conflicting opinions regarding the follow-up of untreated PAVM. Current recommendations for CT follow-up diverge and specify CT radiological time intervals of 3–5 years. Due to the often gradual changes, others suggest an interval of 5–10 years (32, 33). In contrast, for follow-up of surgically or interventionaly treated PAVM, a CT check 6 months after treatment is recommended. Thereafter, CT control should be performed every 3–5 years or earlier if patients have clinical symptoms (21). The role of MRI and TTCE in follow-up is supported by recent studies showing diagnostic accuracy combined with the advantage of less radiation exposure and potential renal damage from contrast agents (3, 34–36). Given our patient's young age, radiological follow-up with TTCE and MRI was recommended after the last discharge.

## 4 Conclusion

PAVM is a rare disorder, and the challenges associated with early diagnosis and selecting the most effective treatment underscore the complexity of managing this pathology. This case illustrates the insidious nature of the condition, where even a preventive, multidisciplinary approach was insufficient to prevent serious complications.

## Data Availability

The original contributions presented in the study are included in the article/supplementary material, further inquiries can be directed to the corresponding author.
